# Geometric Heterogeneity of Continental Shale in the Yanchang Formation, Southern Ordos Basin, China

**DOI:** 10.1038/s41598-017-05144-z

**Published:** 2017-07-20

**Authors:** Lihui Li, Beixiu Huang, Yufang Tan, Xiaolong Deng, Yanyan Li, Hu Zheng

**Affiliations:** 1 Key Laboratory of Shale Gas and Geoengineering, Institute of Geology and Geophysics, Chinese Academy of Sciences, Beijing, 100029 China; 2 College of Earth Science, University of Chinese Academy of Sciences, Beijing, 100049 China; 3 School of Earth Science and Engineering, Hohai University, Nanjing Jiangsu, 211100 China

## Abstract

Favorable prospects for the exploration of shale gas have been demonstrated in the Ordos Basin, China. Outcrop and core observations indicate that there are abundant laminas in the shale strata, which exert a great influence on hydro-fracture propagation, gas storage and fluid flow. In this study, the continental shale of the Chang 72 Member, collected from the south of Ordos Basin, was investigated to characterize the geometric heterogeneity. Laminas at multiple scales were observed and measured using conventional logging, borehole TV, core analysis, scanning electron microscopy, and the Particle and Crack Analysis System. These measurement tools correspond to the meter scale, decimeter scale, centimeter scale, millimeter scale and ten-micrometer scale, respectively, with measured thicknesses of 2.26 m, 2.09 dm, 1.70 cm, 1.48 mm and 11.70 μm, respectively. Fractal theory was used to analyze the power exponent distribution of the lamina thickness, with a resulting fractal dimension of 1.06. Finally, a geometric heterogeneity model was proposed for the Upper Triassic Yanchang Formation in the study area and verified by a modeled thickness of 26.30 m for the Chang 72 Member at the 10-m scale. The model facilitates cross-scale analysis and provides parameter guidance for heterogeneity characterization in the numerical simulation and model test of the shale gas reservoir.

## Introduction

Recently, inspired by the successful exploration and commercial production of shale gas in the USA, many countries and regions have conducted investigations to evaluate shale gas production^1–4^. Organic-rich shale is deposited in both marine environments and non-marine environments: marine-continental transitional environments and continental environments^5, 6^. The typical shale formations in North America are mainly deposited in marine environments: the Barnett Shale in the Fort Worth Basin^3, 7^, the Eagle Ford Shale in the West Texas Basin^8, 9^, the Haynesville Shale in the North Louisiana Salt Basin^7, 10^, the Marcellus Shale in the Appalachian Basin^10–12^, and the Fayetteville Shale^9, 13^ and the Woodford Shale in the Arkoma Basin^10, 14^. A minority of oil shale is formed in lacustrine environments, such as the Green River Formation of Colorado and Utah^15^ and the Wilkins Peak Member of the Green River Shale in Wyoming^16–18^. By contrast, the marine organic shale in China accounts for only 1/3 of all recoverable shale gas resources, such as the Longmaxi Formation and Qiongzhusi Formation in the Sichuan Basin^5, 6^. Moreover, approximately 2/3 of this shale gas is discovered in marine-continental transitional facies and continental facies^19^, for instance, the Xujiahe Formation in the Sichuan Basin and the Shanxi Formation and Yanchang Formation in the Ordos Basin^20^. To date, preliminary investigations on the continental shale of the Ordos Basin have estimated the reserves of gas to be 677 × 108 m³ ^21^, indicating a favorable geological condition for shale gas accumulation and exploration.

Similar to a conventional sandstone reservoir, a shale reservoir is heterogeneous^22^. Influenced by the local climate, the deposition, diagenesis and tectonization in forming the shale reservoir varies, leading to its inhomogeneous characteristics in the spatial distribution and internal properties^23, 24^. Due to the complex and substantial tectonic movements, the shale gas reservoirs in China are characterized by strong deformation and erosion, well-developed fractures and faults, and high thermal maturity and considerable heterogeneity, in particular continental shale. In the shale fracturing process, heterogeneity is one of the main challenges influencing the prediction of productivity^23–29^. Based on geochemistry, petrology and sedimentology analyses, the vertical heterogeneity of the Lower Silurian Longmaxi marine shale can be suggested by its lithofacies, graptolite species and abundance, mineralogy, sedimentary structure, fracture, total organic carbon (TOC) and gas content^28^.

In continental shale reservoirs, the heterogeneity is mainly characterized by the sharp variation of the TOC content, capability of hydrocarbon generation and expulsion, pore structure and mechanical parameters, as well as high density and frequency of developed laminas^22^. Studies of the interbedded layers of shale suggest that sandy laminas are favorable sites for oil and gas to accumulate and for fractures to propagate, providing a migration passage for gas flow^30, 31^. Regarding the continental shale reservoir of the Ordos Basin, studies have shown that as more laminas develop, more free gas and solution gas are contained, resulting in less absorbed gas^32^. This result indicates that the development of laminas has an important effect on gas production. Unfortunately, reports on the geometric heterogeneity of the continental shale in the Yanchang Formation are sparse.

Numerical simulations and models are commonly utilized for investigating the hydraulic fracture behaviors of shale formations^32–44^. Aiming at probing the influence of reservoir heterogeneity on hydro-fractures and optimizing the fracturing design, numerical simulations were performed to simulate the fracture network propagation^45^. The effects of the laminar structure on the hydraulic fracture propagation in shale were also investigated through laboratory models^46^. The multi-layer simulations indicate that there is a distinct retardation in fracture propagation passing the interface of layers with different Young moduli^47^, and a heterogeneous reservoir with multiple layers has a pronounced impact on the fracture geometry and ultimately the production performance^45–47^. It should be noted that in the majority of models, the material heterogeneity used to be characterized with the hypothesis that the properties (failure strength and elastic modulus) of rocks are randomly distributed following a Weibull law^47–51^. However, a gap exists between the randomly generated geological structures in the simulated model and the real geological structures of the reservoir, and it would be more precise to design a model with the in-situ geological structures, including the lithology, real layer thickness, and attitude. To date, few studies on the stratified layer thickness of shale targeted for hydraulic fracturing, as well as on the characterization of laminas at multiple scales, either macroscopic scales or microscopic scales, are available for reference.

In this paper, the geometric heterogeneity of a continental shale gas reservoir is presented through a case study of shale in the Chang 72 Member, Ordos Basin of China. Conventional well logging, digital borehole TV, core observation, SEM scanning and PCAS analysis were performed to characterize the lamina composition and layer thickness at multiple scales. Fractal theory was used to define the geometric heterogeneity from the macroscopic scales to the microscopic scales. The study of the geometric characteristics provides a reliable reference for parameter selection in the simulation and modeling of shale reservoirs, especially for the trans-scale analysis in the Southern Ordos Basin, China.

## Geological settings

**Figure 1:**
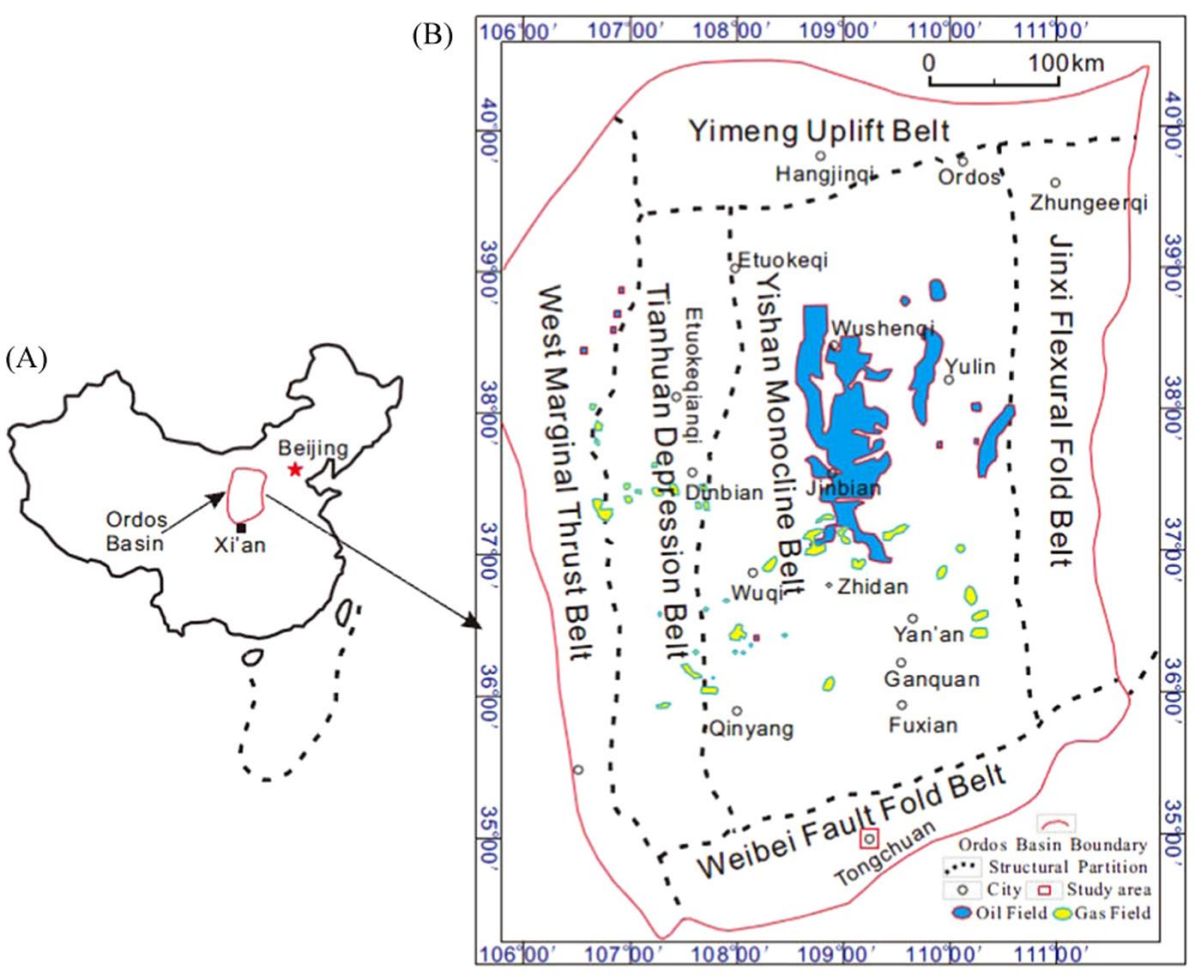
Ordos Basin structure and location of study area. (**A**) Map showing the Ordos Basin in China. (**B**) Tectonic map of Ordos Basin, modified after Lei (2015)^32^; the study area in the south part of the Weibei uplift is outlined by a red box. The source of the map is the Geological Society of America bulletin, Vol. 99(4), April, 2015. Permission for the map was obtained from an Open Access license with license number 4026530233979. The figure was created using the Chinese version of CorelDraw Graphic Suite X8. (http://www.coreldraw.com/cn).

The Ordos Basin is located in the western part of the North China Craton, covering an area of approximately 32 × 104 km^2^. It is a polycyclic superposition basin with vast oil and gas reserves^52, 53^, forming a large asymmetrical syncline with a broad, gently dipping eastern limb and a narrow, steeply dipping western limb. According to the structural evolution and present morphology, the basin is divided into six major structural units: the Yimeng uplift in the north, the Weibei uplift in the south, the central Yishan slope, the Jinxi flexural fold belt in the east, the Tianhuan depression, and the western edge thrust belt in the west (see Fig. 1). The basement of the Ordos Basin, formed in the Archean and Paleoproterozoic, has experienced five evolutionary stages: the aulacogen of the Meso-Neoproterozoic, the shallow marine platform of the Early Paleozoic, the stand plain of the Late Paleozoic, the inland depression of the Mesozoic, and the fault depression of the Cenozoic^32^. Due to the Late Triassic tectogenesis, a large-scale inland freshwater lake was formed in the internal part of this basin, in which the Yanchang Formation was deposited. Subsequent tectonic activities from the late Triassic to the early Cretaceous profoundly affected the generation, migration and accumulation of hydrocarbons. The main source units of this basin is the Upper Paleozoic strata represented by the Carboniferous-Permian coal seams^52^ and the continental shale in the Yanchang Formation of the Upper Triassic strata^53^.

**Figure 2:**
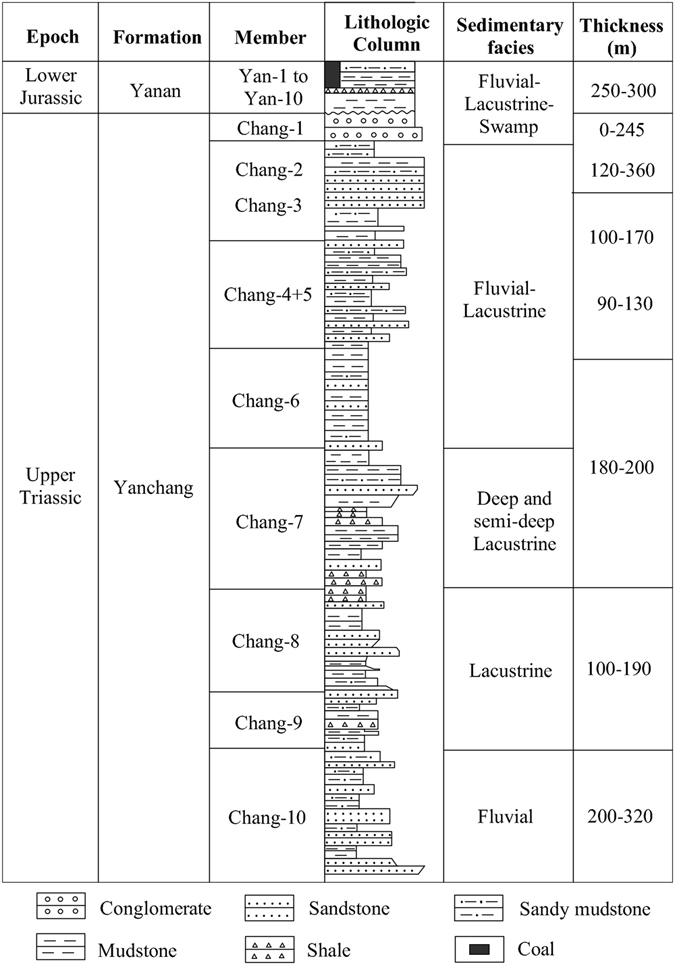
Stratigraphic column of the Yanchang Formation and its sub-members (modified after Guo (2014)^4^). The source of the map is Marine and Petroleum Geology, Vol. 57, May, 2015. Permission for the map was obtained from an Open Access license with license number 4103990262253. The figure was created using the Chinese version of CorelDraw Graphic Suite X8. (http://www.coreldraw.com/cn) 57: 509–520.

Based on the evolution of inland lake in the late Triassic, the Yanchang Formation can be divided into 10 Members (Fig. 2). The lowest Chang 10 Member was deposited in the initial stage of the lake and overlaid by the Chang 9 and 8 Members during the period of major transgression when thermal subsidence occurred. The Chang 7 Member was formed with rapid subsidence, and the water depth reached 50~120 m at the peak stage of significant lake expansion^54^. The Chang 4~6 Members marked an episode of constructive deltaic infilling with a decreasing subsidence rate. The Chang 1~3 Members were mainly deposited during a period of major contraction, and the lake disappeared gradually with the deposition of the Chang 1 Member, dominated by a swamp environment. The Chang 9 and 7 Members have long been recognized as the main high-quality source rock and the key strata with abundant shale gas.

During the depositional stage of the Chang 7 Member, the tectonic movements were relatively active due to the Indosinian tectonic movement in the northwestern area of the basin, causing obvious regularity in the changing of the sedimentary facies. According to the cycle of sedimentation, the Chang 7 Member can be subdivided into three members: Member 71, Member 72 and Member 73. During the episode of the Chang 73 Member deposition, the lake reached its largest area, with less developed turbidite sandstone of semi-deep and deep lacustrine facies. During the depositional period of the Chang 72 Member, the area of the semi-deep and deep lacustrine facies decreased dramatically, with a relatively well developed delta-front sand-body and turbidite sandstone in the phase of the semi-deep and deep lake. In the last Member 71 stage, the lake decreased further, leading to the well development of the delta-front sand-body and turbidite sandstone of the semi-deep and deep lake facies^55^.

**Figure 3:**
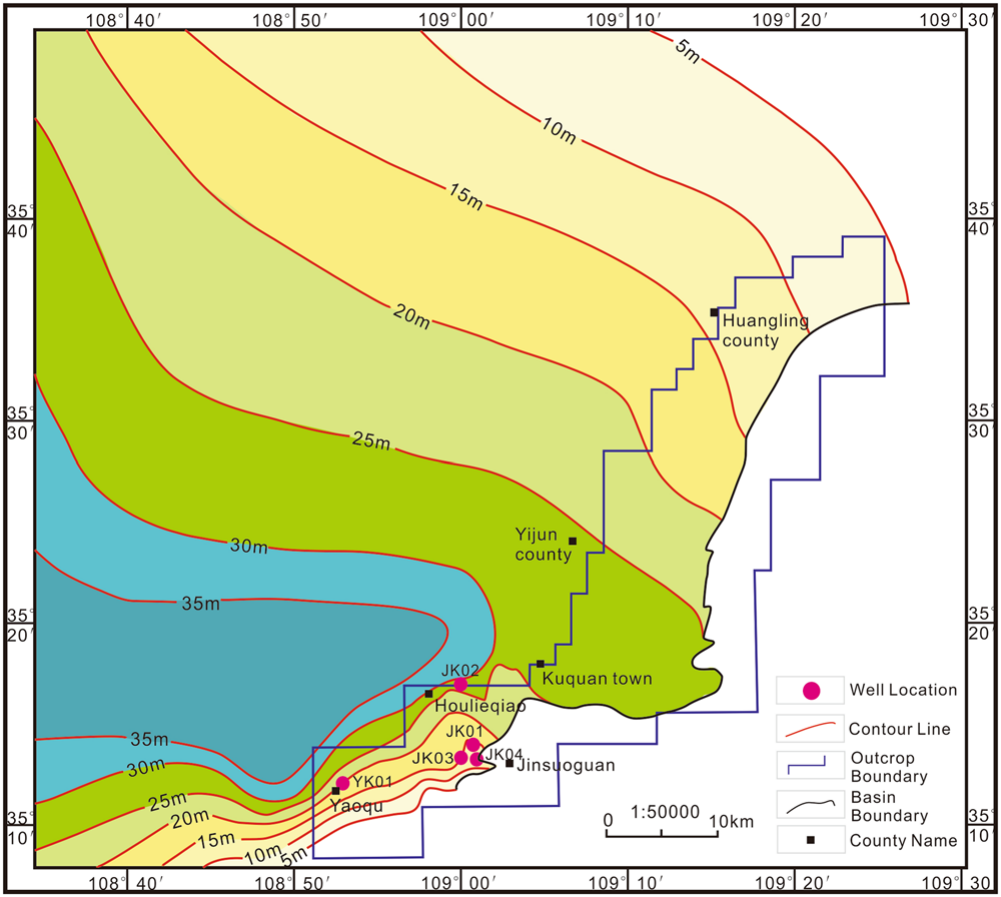
Oil shale thickness distribution of the Yanchang Formation in southeast Ordos Basin, The figure was created using the Chinese version of CorelDraw Graphic Suite X8. (http://www.coreldraw.com/cn).

The outcrops of shale mainly distribute in the south of the Ordos Basin. The geographic position is located in the Yijun-Xunyi-Binxian belt (Fig. 3). The strata thickness of the oil shale outcrops in the south-east of the basin is typically 10–25 meters, and it decreases along the north-east and south-west directions, with the thickest stratum outcrops in the Tang Nihe-Mazhuang area^56–58^.

The study area is located in the southern Weibei uplift of the Ordos Basin, an area where there is generally a lack of detailed information on the lamina characteristics^22, 32, 59^. The focus is on the Chang 7 Member of the Upper Triassic Yanchang Formation. The deposits of the Chang 7 Member consist of deep and semi-deep lacustrine oil shale facies, which are mostly black or blackish gray with elevated organic carbon contents, and they are one of the main source rocks in the study area. The thickness of the Yanchang Formation ranges from 900 to 1600 m, with the Chang 7 member burial depth being 500–2000 m^25^.

## Results

### Mineral composition of laminas

**Figure 4:**
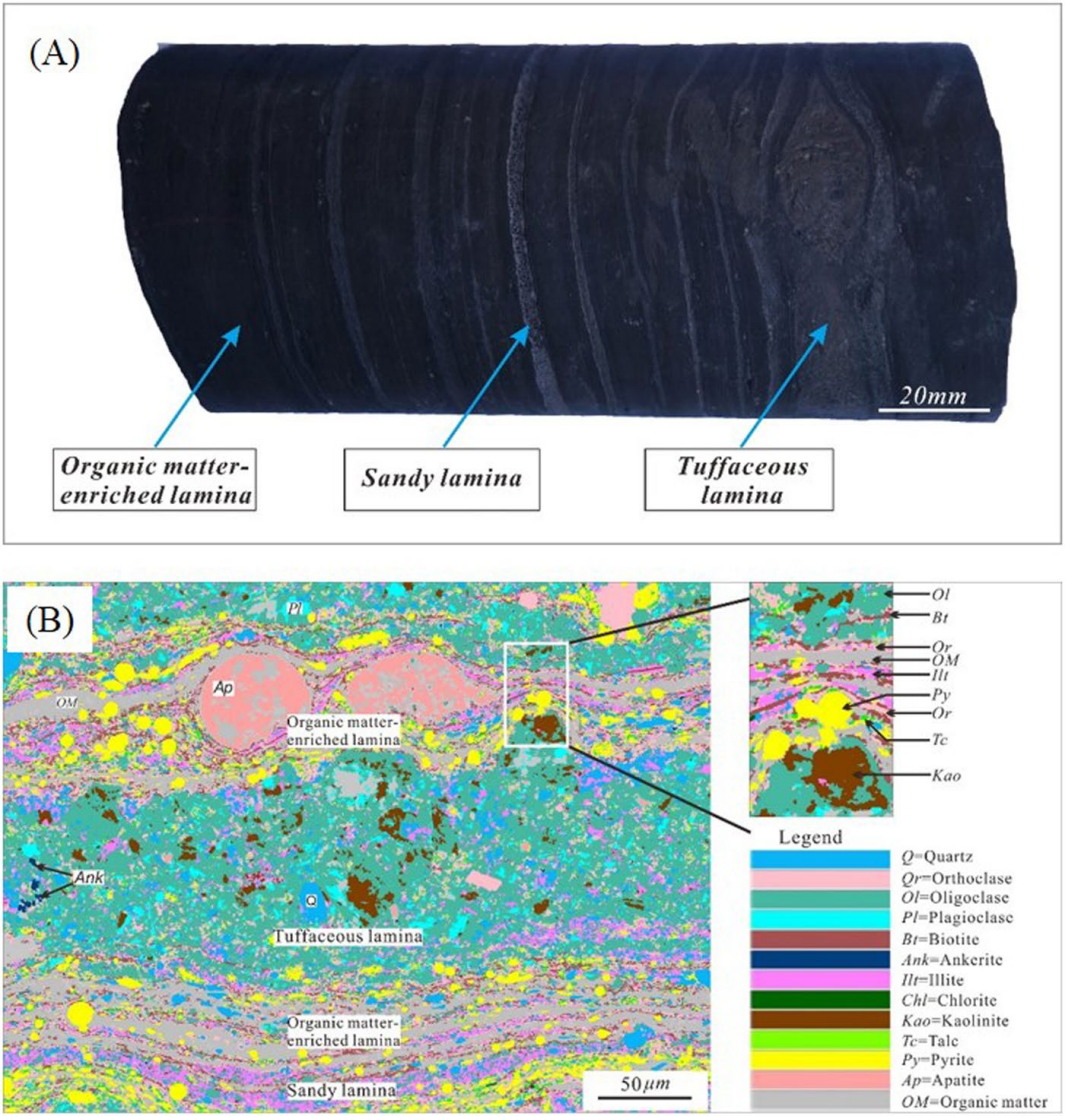
Laminas with different compositions of minerals. (**A**) The sandy lamina is grayish-white, the tuffaceous lamina is brown, and the organic matter-enriched lamina is black. (**B**) BSE image of mineral analysis of shale from the YK01 well. Oligoclase is dominant, followed by orthoclase, pyrite, organic matter, and quartz. The white box amplified on the right indicates that the minerals in the shale have a layered distribution.

The laminas have different compositions of minerals, including sandy lamina, tuffaceous lamina, and organic matter-enriched lamina (Fig. 4A). The sandy lamina is commonly grayish-white. The grain diameters are 0.05–0.25 mm, with a few being 0.01–0.05 mm. However, the pyroclastic grains in the brown tuffaceous lamina are commonly less than 0.05 mm in size. Figure 4B shows a BSE image of the mineral analysis of shale from well YK01. It is noted that the minerals have a layered distribution, in accordance with the deposition of the lamina. In the sandy lamina, there is 12% quartz, 20% feldspar and 16.8% pyrite. However, the tuffaceous lamina contains 9% quartz, 12% feldspar, and 5% pyrite, with 8% calcite and 55% analcite. The content of clay minerals, such as the illite and illite smectite mixed layer, is approximately 51% in the sandy lamina yet only 12% in the tuffaceous lamina.

### Multi-scale thickness of laminas

**Figure 5:**
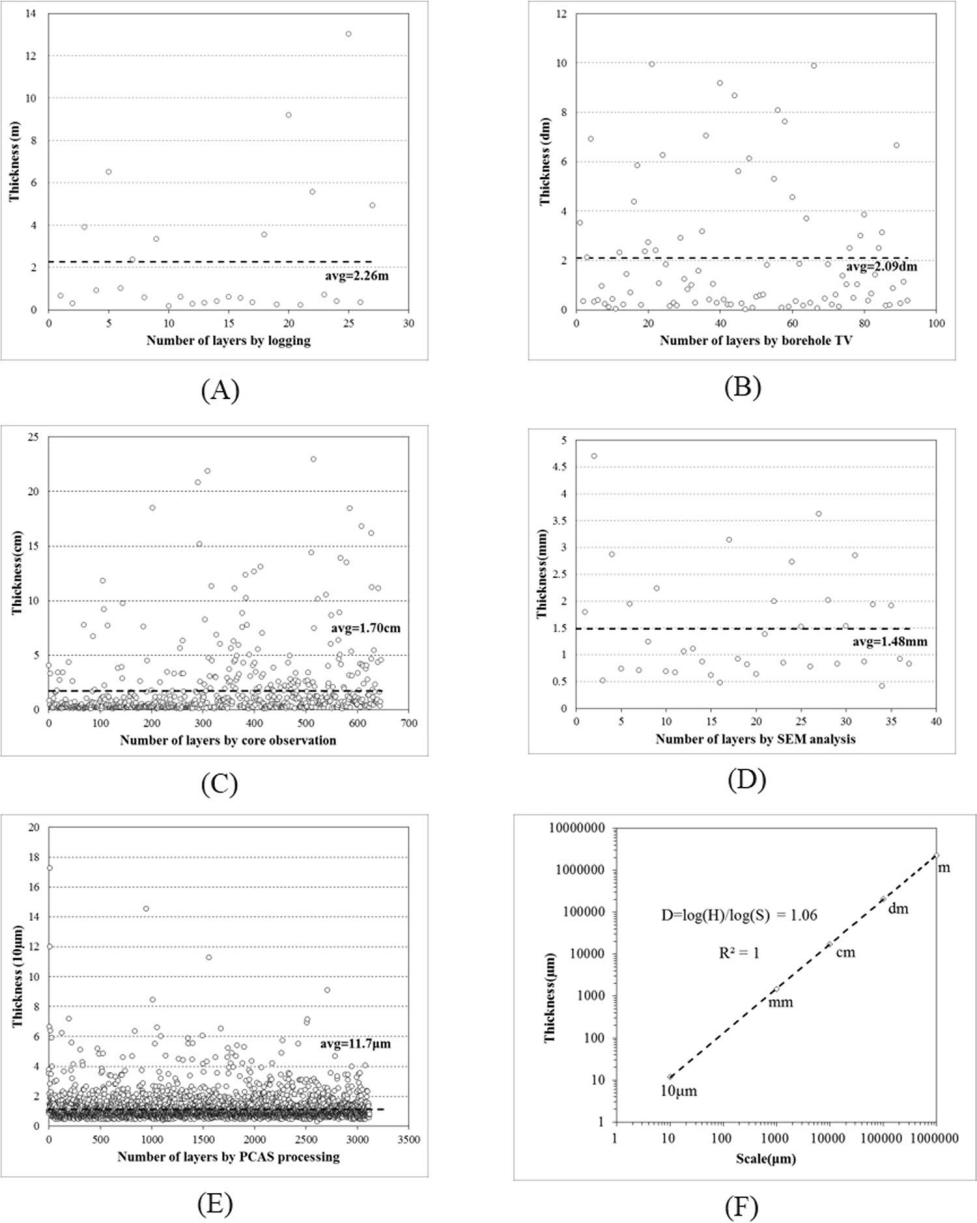
Multi-scale thicknesses and fractal features of the Chang 7 Member shale. (**A**) Thickness distribution of laminas stratified by logging curves at the meter scale. (**B**) Thickness distribution of layers stratified by borehole TV at the decimeter scale. (**C**) Thickness distribution of layers stratified by core observation at the centimeter scale. (**D**) Thickness distribution of layers stratified by SEM image processing at the millimeter scale. (**E**) Layer thickness statistics by PCAS processing at the micrometer scale. (**F**) Double-logarithmic curve of layer thickness and measurement scale.

According to the logging interpretation, 27 layers are stratified in the 60.95-m thick Chang 72 Member shale in the JK01, JK03 and JK04 wells, with an average thickness of 2.26 m for an individual lamina at the meter scale (see Fig. 5A). The total measured thickness of the Chang 72 Member by borehole TV is 18.8 m, including 90 layers, with an average thickness of 2.09 dm for an individual lamina at the decimeter scale (see Fig. 5B). On the basis of the core analysis, 636 laminas are stratified in 10.8-m long cores from the Chang 72 Member, with an average thickness of 1.70 cm for an individual lamina at the centimeter scale (see Fig. 5C). The total measured core in the SEM images is 54.76-mm thick, which was stratified into 37 layers. Therefore, the mean thickness of an individual lamina is approximately 1.48 mm at the millimeter scale (see Fig. 5D). With the PCAS program, 3120 objects were identified in a scene of 36.504 mm width, with an average thickness of approximately 11.70 μm for an individual lamina at the micrometer scale (see Fig. 5E).

### Fractal features of laminas' thickness

The average thickness of an individual lamina measured at the five measured scales (i.e., meter scale, decimeter scale, centimeter scale, millimeter scale and micron scale) was 2.26 m, 2.09 dm, 1.70 cm, 1.48 mm and 11.70 μm, respectively. Previous studies of the thickness characteristics of sedimentary formation imply that the layer thickness has a power exponent distribution^59, 60^, indicating a power exponential relation between the measurement scale and lamina thickness. Herein, a self-similar fractal dimension was applied to describe the relation:

1 $$D=\,\mathrm{log}(H)/\mathrm{log}(S)$$

2 $$H={(S)}^{D}$$

where D is the fractal dimension, H is the layer thickness, and S is the scale size.

Taking the micrometer (μm) as the minimum unit scale, the scale sizes of these five scales are 10 μm, 1000 μm, 10000 μm, 100000 μm, and 1000000 μm, corresponding to the tens of micrometer scale, millimeter scale, centimeter scale, decimeter scale, and meter scale. The measured layer thicknesses at the increasing scales are 11.70 μm, 1479.97 μm, 16986.89 μm, 208977.78 μm, and 2260000.00 μm, respectively. A relation curve of the average layer thickness and corresponding scale is plotted in double-logarithmic coordinates, as shown in Fig. 5F, which is described as follows:

3 $$D=\,\mathrm{log}(H)/\mathrm{log}(S)=1.06$$

Then, Eq. (3) can be changed into a power function form represented by

4 $$H={S}^{1.06}(\mu m)$$

where 1.06 is the fractal dimension.

Table 1 shows an estimation of the layer thickness at multi-scales, which indicates that the estimated thicknesses of the layers are 11.53 μm, 1.52 mm, 1.74 cm, 2.00 dm, and 2.29 m. The errors of the calculated model are 1.48%, 2.48%, 2.42%, 4.50%, and 1.30%, respectively. A good agreement is found between the modeled and measured thicknesses.

**Table 1:** Modeled and measured thicknesses of layers at multi-scales.

Unit	Scale/μm	Measured thickness	Modeled thickness	Error (%)
10 μm	10	1.170	1.153	1.482
mm	1000	1.480	1.517	2.482
cm	10000	1.699	1.740	2.420
dm	100000	2.090	1.996	4.501
m	1000000	2.260	2.289	1.296

## Discussion

The power exponent of 1.06 for the lamina thickness in the Chang 72 Member indicates that the layer thickness has a unified fractal feature over multiple scales (meter scale, decimeter scale, centimeter scale, millimeter scale and micron scale). This result suggests that the shale reservoir is well developed in laminas at all sample sizes, and the layer thickness at different scales could be estimated with analyzed fractal dimensions.

To verify the accuracy of the proposed model, the unit of the formula was extrapolated to the ten-meter scale (basin scale). The modeled individual lamina corresponds to the Chang 72 Member, which yields a 26.30-m thickness at the ten-meter scale, in agreement with the investigated thickness (15 m–30 m) of the Chang 72 Member in Yanchang Formation, Ordos Basin^57, 61^. The verification suggests that it is possible to estimate the layer thicknesses of various scales with fractal formula (3) or (4). This provides an access to a geometric parameter reference for physical or numerical model design, and enables the trans-scale analysis of shale reservoirs in the Southern Ordos Basin, China.

At present, the optimization of the propagation of hydraulic fracture networks is complicated and challenging. Many field observation methods and laboratory experiments have been performed to investigate the fracture propagation^42–44^. Meanwhile, numerical modeling is preferable owing to its flexibility in being able to simulate field conditions. It also has the advantage of taking into account of more details for analysis. However, it is difficult to establish proper models in consideration of all the important factors, including natural fractures, rock properties, in situ stress, fluid properties, injection rate, and injection of proppant. Usually, numerical analysis is based on highly ideal assumptions, for example, infinite layer thickness for a single fracture and a homogeneous and isotropous rock matrix^44, 62^. According to a comprehensive analysis of concrete samples with properties similar to the continental shale, the cracks have the greatest extent and the strongest impact on hydraulic fracturing when a similar lamina in the shale is 4 cm thick^63^.

Therefore, it is important to evaluate the rock properties quantitatively, with accurate input parameters for numerical simulation. Herein, the characterization of the laminar geometric heterogeneity in shale provides guidance for the input of geometric parameters in numerical analysis and reference for model design in the laboratory, in particular for research on the lacustrine shale of the Ordos Basin. With the fitting fractal dimension above, it is possible to extend the size of the lamina to more macroscopic and more microscopic scales, which is key to solving the scale effect and realizing cross-scale analysis.

In conclusion, this work studied the petrographic and geometric heterogeneity in samples of the Chang 7 Member from the Ordos Basin, China, using various measurement tools spanning from the microscopic to macroscopic length scales. The observation, analysis, and interpretation of the lithology and thickness characteristics for multi-scale laminas can be summarized as follows:

The laminas have different compositions of minerals, including grayish-white sandy lamina, brown tuffaceous lamina, and black organic matter-enriched lamina. The content of organic matter in the sandy lamina is low with an irregular distribution, whereas it is higher in the tuffaceous lamina with a dispersive distribution.

The thickness of the lamina in the shale follows a power exponent distribution with a fractal dimension of 1.06. The average thickness of the laminas is approximately 2.26 m, 2.09 dm, 1.70 cm, 1.48 mm and 11.70 μm, corresponding to the meter scale, decimeter scale, centimeter scale, millimeter scale and micron scale, respectively.

The continental shale of the Ordos Basin is highly stratified by laminas, presenting strong heterogeneity in lithology and geometric structure with a length scale effect. The heterogeneity model provides access to cross-scale analysis, which can serve as a reliable reference for parameter selection in the simulation and modeling of shale reservoirs in the Southern Ordos Basin, China.

## Methods

**Figure 6:**
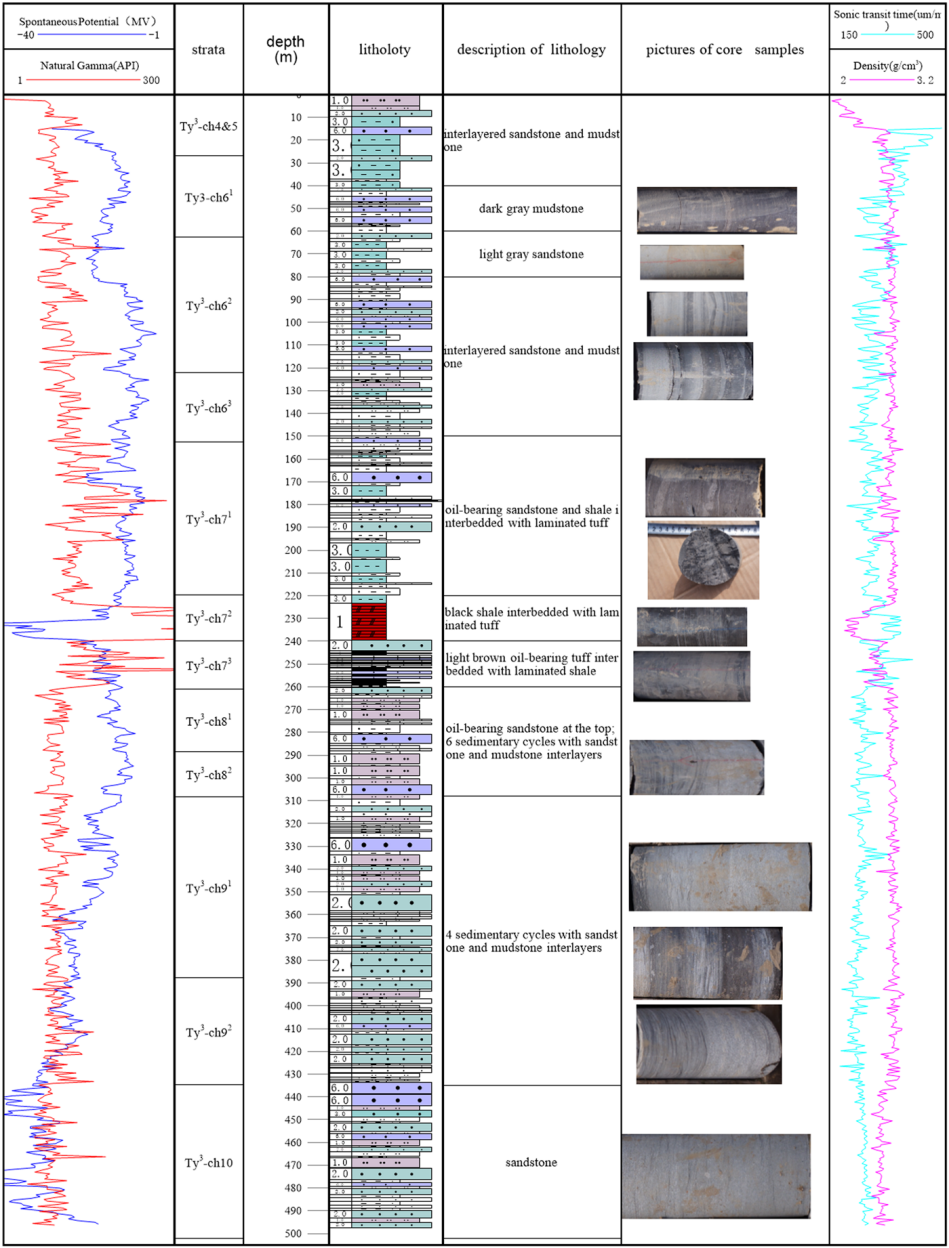
Lithological and electronic characteristics of the Yanchang Formation in well YK01. The photographs of the cores in this figure were taken in well YK01 by the corresponding author. Well YK01 is located in Yaoqu County, Tongchuan City, Shaanxi Province.

To study the heterogeneity of the continental shale, four exploratory wells were drilled; i.e., the YK01 well (located in Yaoqu town of Tongchuan city, with a footage of 502 m) and the JK01~JK04 wells (located in Jin Suoguan town of Tongchuan city, with a total footage of 470 m) were drilled, as shown in Fig. 3. After completion, gamma ray logging (GR), acoustic logging (AC), and resistivity logging (RT) were performed in the YK01 and JK01-JK04 wells. The exposed strata are mainly the Chang 4~10 Members of the Upper Triassic Yanchang Formation. The measured comprehensive stratigraphic diagram is shown in Fig. 6.

**Figure 7:**
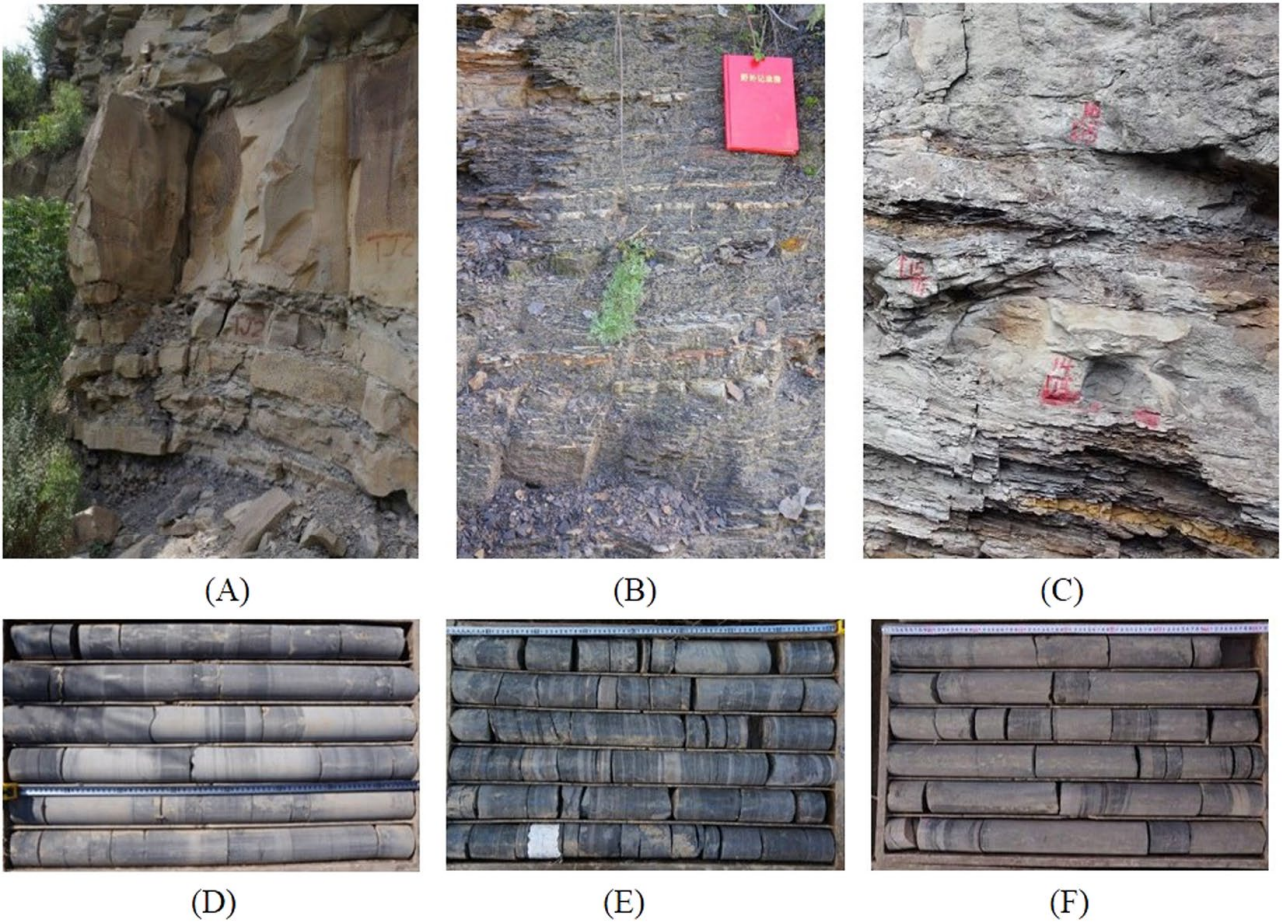
Lithology characteristics of the Chang 7 Member (Yanchang Formation of the Ordos Basin). (**A**) Outcrop of the Chang 71 Member, thick or medium thin sandstone interbedded with thin oil shale. (**B**) Outcrop of the Chang 72 Member, thin oil shale interbedded with thin tuff and sandstone. (**C**) Outcrop of the Chang 73 Member, thick tuff interbedded with thin oil shale and sandstone. (**D**) Core photograph of the Chang 71 Member. (**E**) Core photograph of the Chang 72 Member. (**F**) Core photograph of the Chang 73 Member. The core photographs in this figure were taken in well YK01. The photographs of the outcrops in this figure were taken in Yaoqu County, near well YK01. All of these photographs were taken by the corresponding author.

Typical outcrops of the Yanchang Formation were investigated in the south of the Ordos Basin. Figure 7 shows the outcrops and drilled cores of the Chang 7 Member. Affected by the Indosinian tectonic movement, the three sections of the Chang 7 Member exhibit regularity in their phase changes. According to the observation of outcrops and drilled cores, the lithology of the Chang 71 Member is characterized by thick and medium-thin grayish-white sandstone interbedded with thin black oil shale; the Chang 72 Member represents an important member of oil shale, with a lithology of thin black oil shale, thin brown tuff interlayers and grayish-white sandstone interlayers; and the Chang 73 Member consists of thin black oil shale, thick brown tuff and grayish-white sandstone interlayers. The thickness of the oil shale is approximately 17 m in the Chang 72 Member, whereas the tuff layer containing oil is merely 4 m. By contrast, the tuff layers with oil in the Chang 73 Member are thicker, reaching up to 18 m, whereas the thickness of the oil shale is less than 4 m.

It is well known that the clastic rocks are heterogeneous due to the presence of bedding. Bedding is one of the stratified structures of rock and is the most typical and important characteristic of clastic rocks. It reflects the variance of rock properties in the vertical direction, usually identified with the change of the mineral composition, texture and color. The basic unit of bedding is a lamina that is deposited from sediments with identical rock properties under a certain condition. Usually, the lamina is relatively thin, ranging from several millimeters to several centimeters^64^, corresponding to millimeter to centimeter scales. In the Chang 72 member, a total of 78 laminas (including sandstone and tuff) were identified in a 1 m profile of outcrop, and there were 324 layers in 20 m of the YK01 well cores, with the thickness of a lamina approximately 0.68–20 mm. The maximum thickness is up to 641 mm. The shale strata in the Chang 72 Member were targeted to analyze the spatial characteristics of the lamina thickness variance for the heterogeneity interpretation.

The heterogeneity of the shale gas reservoir depends on the measured scales, with clear scale effects. Therefore, it is necessary to explore the individual features of heterogeneity at the different scales in detail to characterize the heterogeneous reservoir at every scale. It is important to discover the nature of the size-dependent phenomena, i.e., the interaction and effect between different levels. For the description of the heterogeneity, the lamina thickness under different scales is measured with different observation tools. Fractal theory is utilized for the establishment of relations between various scales.

The logging curves were used to measure the meter-scale thickness of the lamina. As shown in Fig. 6, the logging curves indicate the content of radioactive mineral, TOC, high specific resistivity, high natural gamma and high interval transit time. The curves of the resistivity and interval transit time could well be utilized to differentiate oil shale from interbeds.

**Figure 8:**
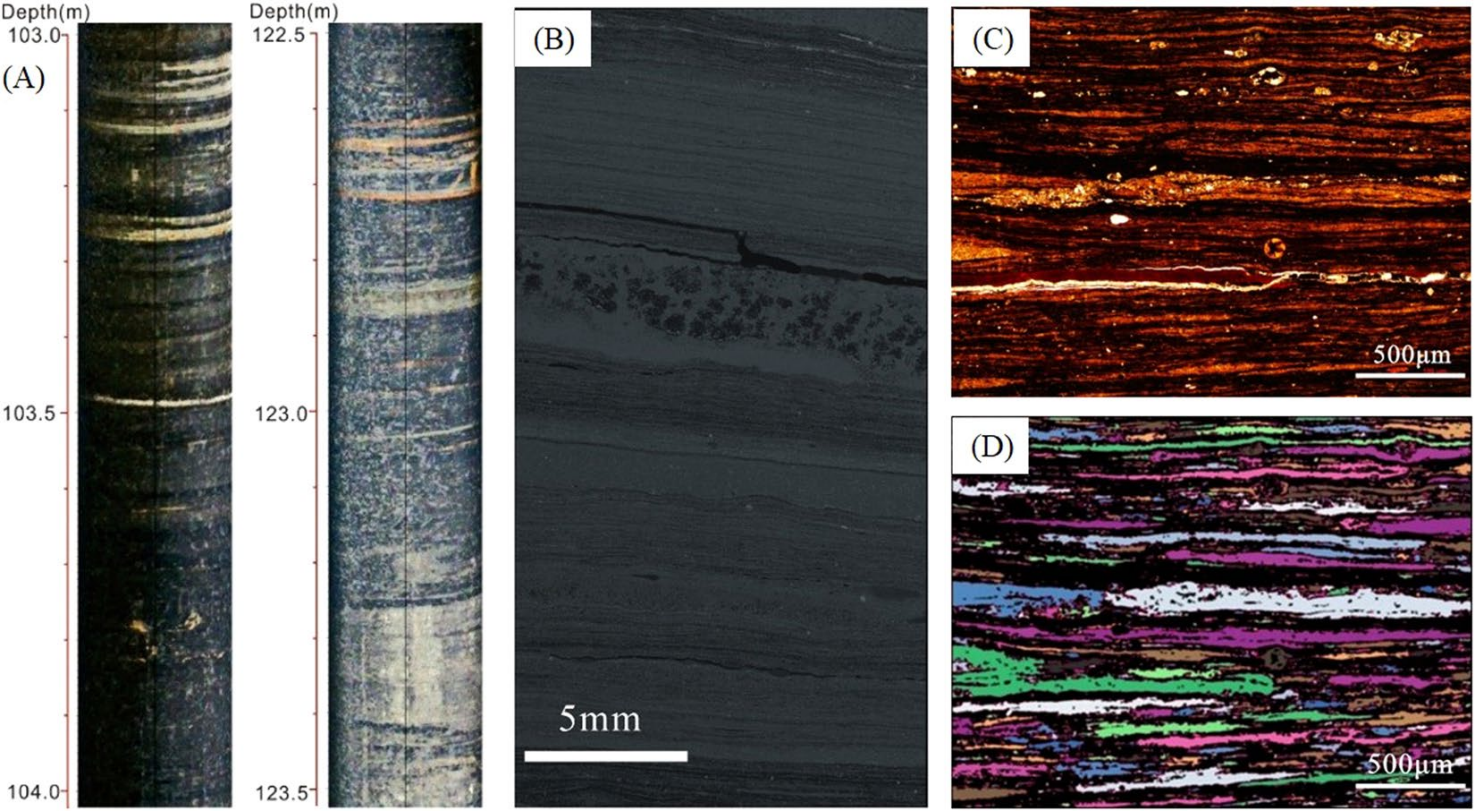
Stratified lamina with various measurements. (**A**) Borehole TV images, where the lamina can clearly be seen. (**B**) SEM image of internal layers in shale of the Chang 7 Member at the millimeter scale. (**C**) Thin-section photomicrographs of laminated shale, showing a variety of sandy laminas with many round apatite concretions. (**D**) Processed color image in PCAS, showing objects being extracted from the pixel matrix.

The borehole TV, core observation, SEM scanning and PCAS system were applied to obtain statistics on the thickness of the laminas on the decimeter, centimeter, millimeter, and 10 micrometer scales, respectively (see Fig. 8). The borehole TV can characterize the discontinuities, tectonic traces, major structural planes and vein orientations. By processing panoramic pictures of boreholes, a virtual core graph was obtained, as shown in Fig. 8A, from which the extensive emergence of thick shale layers could be observed below a depth of approximately 103 m. A core analysis was performed by observing rock cores from the Chang 72 Member of the Yanchang Formation to characterize the lithology, sedimentary structures, geometry, and types of lamina at the centimeter scale. A caliper with a precision of 0.02 mm was used to measure the thickness of the individual lamina by eye. The number and thickness of the individual lamina were documented. With the aid of the Scanning Electronic Microscope (SEM) technique, images of the laminas on the millimeter scale were obtained to provide statistics based on their thickness (Fig. 8B). Using Image Pro-Plus 5.0 software, the image intensity grade is best fit to enhance the image contrast and improve the display effect. The layer morphology and petrography are utilized to differentiate the shale layers from sandy and tuffaceous interbeds.

The Particle (Pore) and Crack Analysis System (PCAS)^65, 66^ was adopted to quantify the thickness of the lamina at the 10 micrometer scale. The scene in a digital image is stored as a matrix of pixels that can be regarded as dots or small squares. Subsequently, objects in the scene can be extracted from the pixel matrix by the application of image processing techniques. Thin-section photomicrographs taken from shale in the Chang 72 Member were imported into the PCAS program to provide stratification and thickness statistics of objects in the scene (as shown in Fig. 8C,D). The segmentation threshold was adjusted with human-computer operation, with a minimum scene area of 50 pixels.
